# Flexible color perception depending on the shape and positioning of achromatic contours

**DOI:** 10.3389/fpsyg.2015.00620

**Published:** 2015-05-18

**Authors:** Mark Vergeer, Stuart Anstis, Rob van Lier

**Affiliations:** ^1^Laboratory of Experimental Psychology, KU LeuvenLeuven, Belgium; ^2^Department of Psychology, University of California, San DiegoSan Diego, CA, USA; ^3^Donders Institute for Brain, Cognition and Behaviour, Radboud University NijmegenNijmegen, Netherlands

**Keywords:** color, filling-in, illusions, shape, contours

## Abstract

In this study, we present several demonstrations of color averaging between luminance boundaries. In each of the demonstrations, different black outlines are superimposed on one and the same colored surface. Whereas perception without these outlines comprises a blurry colored gradient, superimposing the outlines leads to a much clearer binary color percept, with different colors perceived on each side of the boundary. These demonstrations show that the color of the perceived surfaces is flexible, depending on the exact shape of the outlines that define the surface, and that different positioning of the outlines can lead to different, distinct color percepts. We argue that the principle of color averaging described here is crucial for the brain in building a useful model of the distal world, in which differences within object surfaces are perceptually minimized, while differences between surfaces are perceptually enhanced.

## Introduction

Luminance and color play a distinct role in vision and are processed in different subareas of the retino-geniculo-cortical pathway (De Valois et al., 1966; Wiesel and Hubel, 1966). Chromatic signals derive from differences in activity in S-, L-, and M-cones, while activity in the luminance channel is derived from additions of the signals of the different cones. The luminance signal is crucial in shape detection and segmentation, as object boundaries are generally characterized by abrupt luminance changes. Fast and accurate detection and segmentation of different animate and inanimate objects are crucial for a primate's survival. Therefore, such abrupt luminance contrasts are reinforced through lateral inhibition (Kuffler and Nicholls, 1976) by means of which the darker side of a boundary is perceptually darkened while the lighter side is further lightened. Subsequently, achromatic and chromatic signals interact to determine an object's shape and surface appearances (e.g., De Weert and Wade, 1988; Kanga and Shevell, 2008).

Human acuity is much lower for color than for luminance (Wandell, 1995). For instance, you can split a picture into its luminance and color components and blur the color component, followed by reuniting the two components. As a result the picture looks virtually unchanged, and resembles the original picture. This shows that the human visual system has poor acuity (low bandwidth) for color (Livingstone, 2002). Television signals take advantage of this principle in encoding images by devoting less resolution to chromatic information than to luminance information (Winkler et al., 2001). So to summarize, the visual system strongly relies on the luminance signal, more specifically (sharp) luminance changes in the visual scene, to define the boundaries of objects and, hence, their shape.

A number of published studies highlight the interplay between color and luminance information in visual processing. The “watercolor illusion” (Pinna et al., 2001) is a striking demonstration that involves contour dependent color spreading. In their study, a wiggly black line and an additional juxtaposed yellow line enclose an area, causing the whole enclosed area to be perceptually tinged with an apparent pale yellowish tint. The afterimages of watercolor-like displays show similar spreading effects when the outlines are presented sequentially, in an alternating fashion (Hazenberg and Van Lier, 2013). Daw (1962) and Van Lier et al. (2009) have demonstrated the role of luminance contours in the appearance of colored afterimages. Colored afterimages are due to adaptation of retinal cones and they are especially vivid when contours, presented after the adapting image, coincide with the blurred edges of the afterimage (Daw). Van Lier et al. demonstrated that weak, blurry color signals from afterimages could spread within regions defined by strong luminance borders but could not cross over these borders. Thus, one and the same colored stimulus can induce multiple, differently colored afterimages, depending on the test contours presented after the colored image. Anstis et al. (2012a) went on to show that the color-contour interactions shown for afterimage colors also occur for “real” colors. They argued that for both types of stimulation the color signals spread by a process analogous to physical diffusion, until they encounter a strong contour such as a black line. In a similar vein, Anstis et al. (2012b) took two pictures, Gainsborough's “Blue Boy” and a pink nude called “La Source” by Ingres. They split each picture into its color and luminance components, and superimposed just the two color components. This gave a rather indistinguishable colored mess. But when the greyscale luminance picture of the Blue Boy was superimposed, the mess looked like the original Blue Boy, and when the greyscale luminance picture of La Source was superimposed, the very same mess looked like the original La Source. Thus, the superimposed luminance contours modulated the perceived colors: the Gainsborough greyscale made the torso look blue, while the Ingres greyscale made the same torso look pink. With regard to the filling-in of afterimages, Francis (2010) and Kim and Francis (2011) explained such contour dependent filling-in effects with a model in which the contour forms a boundary that traps afterimage colors, and presumably real colors, as they spread across a surface. This model in turn draws upon the earlier theories of Grossberg and Mingolla (1985) and Grossberg (2002). These two latter papers also propose that color spreads spatially in a process akin to physical diffusion, until it encounters a luminance contour. The experiments of Kim and Francis (2011) also showed that their model could not fully account for the spreading of afterimage colors.

**Figure 1:**
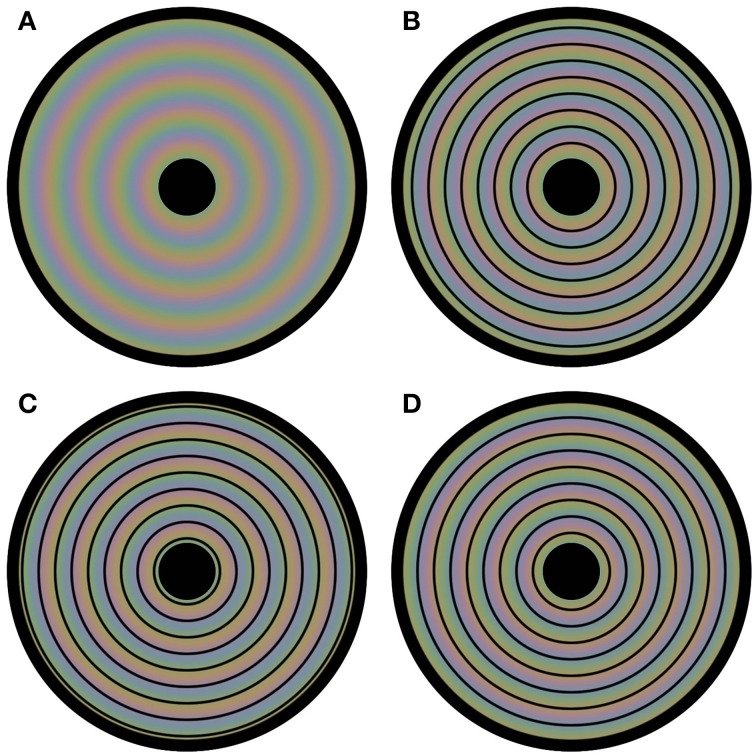
**Flexible colors**. All four panels contain a disk that evokes four distinct colored experiences. However, each panel has the same underlying blurry colored image. The only physical difference between the panels is the positioning of circular outlines on top of this colored image. In **(A)** no outlines are presented, leading to a continuous perceptual color change from the center to the periphery of the disk. The positioning of the contours is at a different phase for each of **(B–D)**. Although the physical color changes are gradual and continuous in all panels, the color appearance seems rather homogeneous between each pair of neighboring outlines; the colors average between the outlines perceptually.

We now present several new demonstrations that further highlight this kind of interplay between luminance and color in visual processing. These demonstrations show that color perception is flexible and depends on luminance based surface construction. We have constructed a novel stimulus by first composing three square-waved, concentric, circular gratings, each consisting of an opponent color pair. The three circular gratings differed 1/3 of a cycle in phase. Next, the three composed images were blurred, superimposed and averaged, with the colored disk in Figure 1A as a result. In fact, in each of the four panels in Figure 1 the same colored disk is displayed, with circular outlines superimposed on the colored disk in Figures 1B–D. The perceptual color appearances are clearly different in all four panels, although, with the exception of the black outlines, the physical color is the same at each location of the disk for all four images. The color gradient visible in Figure 1A, is perceptually absent in the other panels, or at least highly reduced. Instead the presented colors seem to perceptually average, forming steps of more or less uniform color between the presented contours. Apparently, the colors seen depend upon the location of the black outline circles. Video 1 shows a dynamic version of this illusion, in which the contour changes position every second. This Video gives an even clearer impression of the different color percepts for the different color settings.

**Figure 2:**
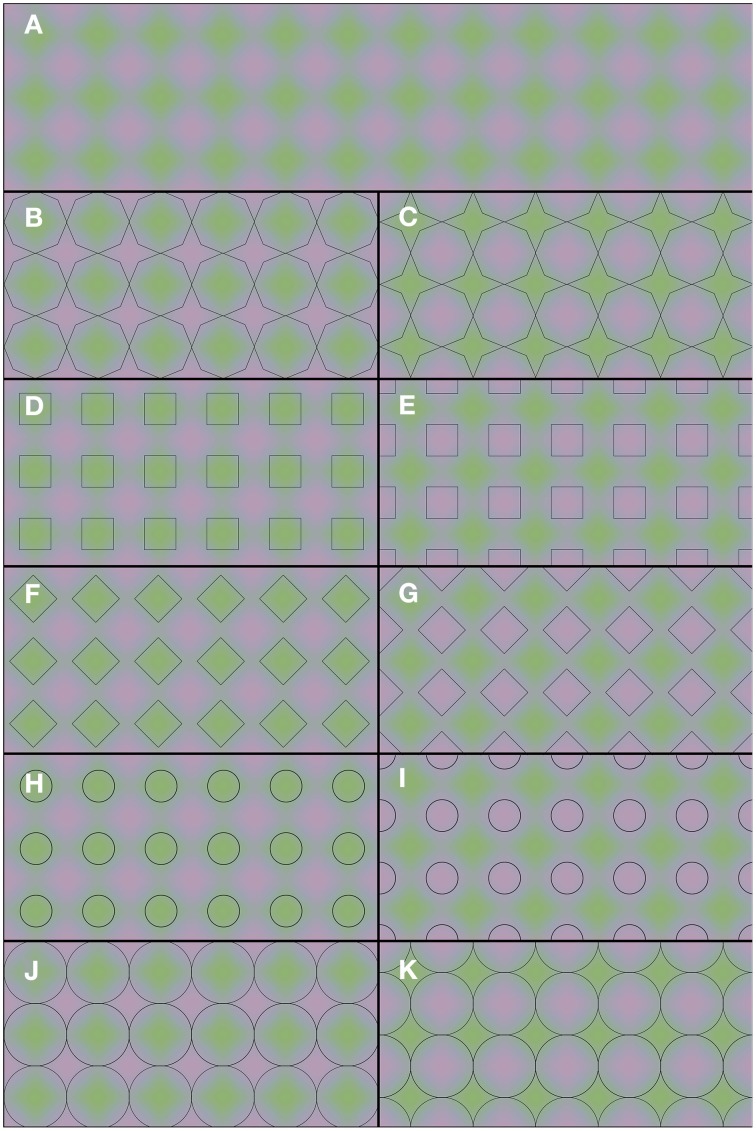
**Flexible color averaging between outlines**. Again, the underlying colored image is the same for each panel. In fact, the total figure is one regular purple-green plaid (as seen in **A**) on which the horizontal and vertical panel dividers and the black outlines within each of (**B–K**) have been superimposed.

The demonstrations in Figure 2 provide a rather different example of color averaging. Again the underlying colors are presented in Figure 2A, and they are repeated throughout the whole figure. Only the superimposed black contours differ between panels. One may note the apparent color differences between the panels. Each panel leads to green and purple color appearances, but the shapes with homogeneous color appearances are perceptually different in each panel. It is the shape and the positioning of the outlines that determine the overall color appearance. Thus, one and the same green and purple colored grid (Figure 2A) can lead to a multitude of different percepts: green octagons and purple stars (Figure 2B), purple octagons and green stars (Figure 2C), green squares (Figure 2D), purple squares (Figure 2E), green diamonds (Figure 2F), purple diamonds (Figure 2G), small green disks (Figure 2H), small purple disks (Figure 2I), large green disks (Figure 2J), and large purple disks (Figure 2K). In Video 2, a dynamic version of Figures 2B,C is shown. In this dynamic version the contour gradually shifts between the 2 contour positions of Figures 2B,C. This Video provides an impression of the dynamic changes in color perception from one contour setting to the other.

**Figure 3:**
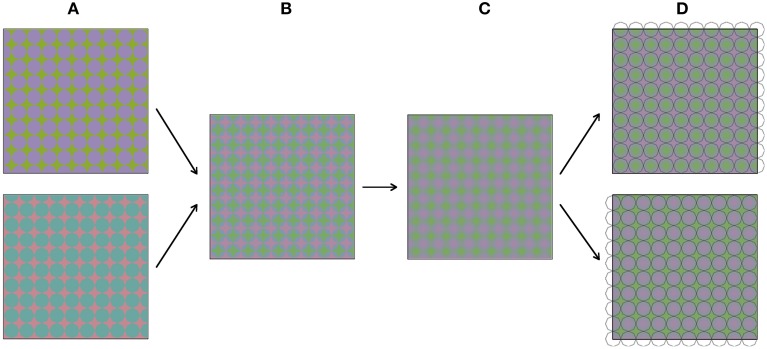
**(A–D)** Visualize sequentially how the stimuli from Figure 2 were constructed.

Figure 3 shows how the stimuli in Figure 2 were constructed. The construction starts with two images (Figure 3A), both comprising a grid of octagons. The two colors in these images were chosen from the so-called Teufel colors (Teufel and Wehrhahn, 2000). This is a set of sixteen colors that are approximately isoluminant, equally detectable and perceptually equidistant. The color pairs in the upper and lower image are orthogonal in color space. The two images are in spatial counter phase, both horizontally and vertically. In the next step (Figure 3B), the two images are superimposed and made semi-transparent, so that a mixture of both images can be perceived. Next (Figure 3C), the image is blurred with a Gaussian blur. Figure 3D shows the result of this process, which is the same colored plaid as displayed in Figure 2A. When luminance outlines are superimposed on the colored plaid, the colors appear to average between these outlines. As was mentioned above, the stimulus was constructed from grids of octagons. Therefore, superimposing octagon-shaped black outlines most likely leads to the most vivid color experience. However, when differently shaped outlines are superimposed on the colored image (see Figures 2D–K), the color also average perceptually within the outlined areas.

In two experiments, to be discussed next, we have tested these effects of color spreading. In Experiment 1 colors were compared and in Experiment 2 colors were matched.

### Experiment 1: a comparison task

#### Participants

Eight naïve observers (6 females), all with normal color vision were tested in this first experiment. The ethical committee of the Psychology Department of the University of Leuven approved both experiments.

#### Apparatus

The experiment was run on a 13 inch MacBook Air (mid 2011 edition), with a screen resolution of 1440 × 900, driven by a 1.7 GHz Intel Core i5 processor at 60 Hz. Images were displayed using Powerpoint for Mac 2011.

#### Stimulus materials and procedure

**Figure 4:**
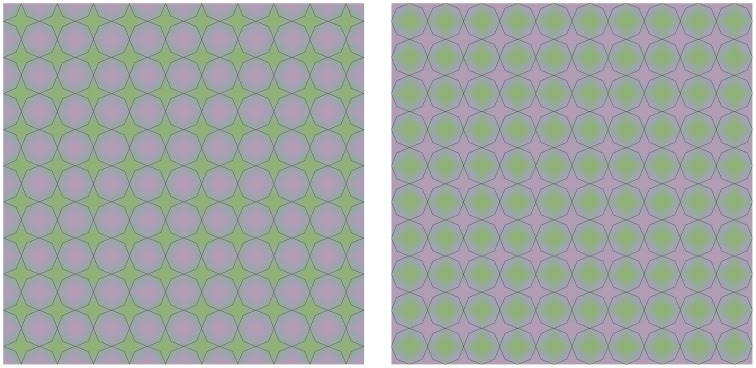
**Example of a trial**. Two identical color images were presented on the left and the right side of the screen in each individual trial. The superimposed outlines were also identical, but could either be positioned in phase, or in counter phase (as in the example above.) Observers had to indicate if the color appearance in both images was identical or not.

As stimulus material we used images based on Figures 1, 2. In each trial, 2 images were presented side by side, always with the same underlying colors for the left and right side image. The overlying contours on the left and right side images could be positioned either similarly (same contour trials) or out of phase (different contour trials). In trials where the circular images from Figure 1 were tested, each circle had a diameter of 10.8 arcdeg. The 2 images were presented on a homogenous white background (L = 340 cd/m^2^). Black outlines had a width of 2.15 arcmin. The stimuli of Figure 2 were presented in a square configuration (diameter 10.8 × 10.8 arcdeg) with 9.5 repeated cycles per image, both horizontally as vertically. The superimposed black outlines had a diameter of 0.86 arcmin. The colors in this image ranged from green (CIExy = 0.329, 0.412; L = 126 cd/m^2^) and purple (CIExy = 0.310, 0.306; L = 126 cd/m^2^). In the different contour trials, observers compared each combination of different contoured images of Figure 1B vs. Figure 1C, Figure 1B vs. Figure 1D, Figure 1C vs. Figure 1D and each contour version of Figure 2 vs. its counter phase alternative (Figure 2B vs. Figure 2C, Figure 2D vs. Figure 2E, Figure 2F vs. Figure 2G, Figure 2H vs. Figure 2I, and Figure 2J vs. Figure 2K). All comparisons were randomized and repeated 4 times for each observer. The observer's viewing distance was approximately 40 cm and his/her task was to indicate whether the colors of the left display and the right display were the same or different. The observer reported their response verbally, by saying either “same” or “different and the experimenter logged the answer. The observer then continued to the next trial by pressing the space bar. An example of a trial is displayed in Figure 4.

## Results

All observers were fully consistent in their choices. In all same contour trials, all eight observers indicated that the color appearance was the same (i.e., 100% “same” judgments). In all the different contour trials, the observers indicated the overall color appearance to be different (i.e., 100% “different” judgments). That is, a bit surprisingly perhaps, there was no variation at all. This result indicates the robustness of the effect of color averaging across observers^1^.

### Experiment 2: a color matching task

The aim of this experiment was to quantify the strength of the effects demonstrated in Figure 1.

#### Participants

Five observers (all males), all with normal color vision, two of whom are authors on this paper, participated in this experiment.

#### Apparatus

The same computer was used as in Experiment 1. Stimulus presentation, timing and keyboard responses were controlled with custom software programmed in Python 2.7 using the PsychoPy library.

#### Stimulus materials and procedure

**Figure 5:**
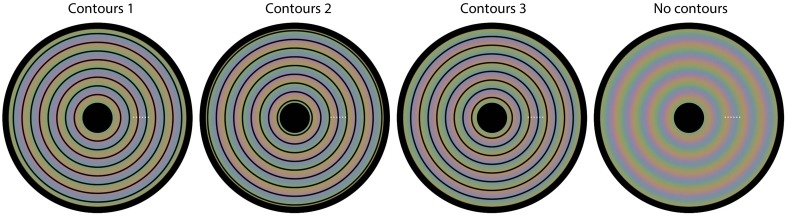
**The four images used in Experiment 2**. Superimposed on each image, we have plotted six small white dots. In each trial, one of the six dots was presented, indicating the location of the color that needed to be matched on that trial. Each different setting was repeated three times.

We used a matching task for this quantification. In each trial of the experiment, one of the four images from Figure 1 was presented (diameter 19.8 arcdeg), with a white dot superimposed at one of six possible locations. The dot locations were equidistant and the same for each of the four images (see Figure 5 for the possible dot locations). The colors at the 6 possible dot locations were as follows: (1) CIExy = 0.364, 0.390, L = 96.1 cd/m^2^; (2) CIExy = 0.370, 0.373, L = 93.4 cd/m^2^; (3) CIExy = 0.325, 0.326, L = 97.7 cd/m^2^; (4) CIExy = 0.285, 0.303, L = 90.2 cd/m^2^; (5) CIExy = 0.275, 0.318, L = 97.4 cd/m^2^; (6) CIExy = 0.312, 0.371, L = 95.3 cd/m^2^. The task for the observer was to adjust the color of a matching gray disk with a diameter of 1.43 arcdeg (initial L = 87.8 cd/m^2^), presented below the image superimposed on a larger, constant gray disk with a diameter of 3.86 arcdeg (L = 87.8 cd/m^2^), until the color of the inner disk was perceptually similar to the color exactly surrounding the presented white dot. Observers adjusted the RGB values of the, initially, gray inner disk by pressing keyboard buttons. The observers were explicitly instructed to match the color directly surrounding the white colored small dot.

## Results

**Figure 6:**
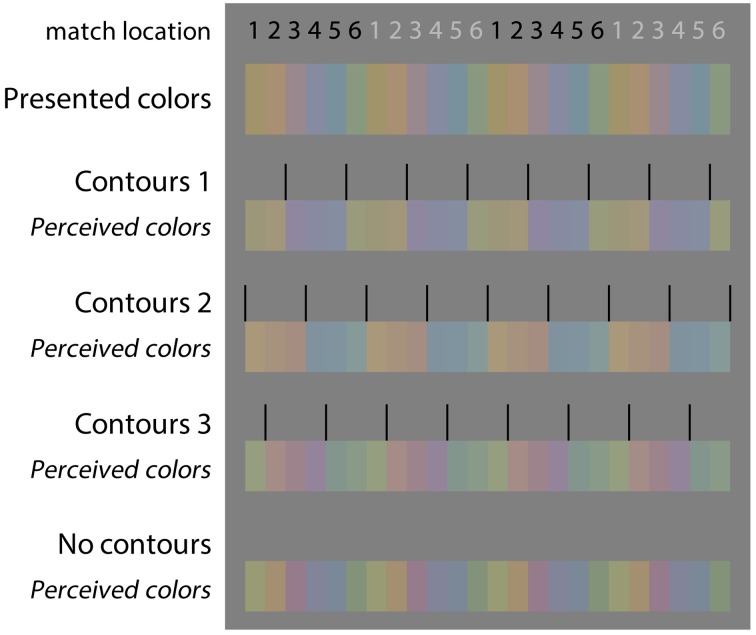
**Color matching results**. For each of the images of Figure 5, the presented color, the positioning of the outlines (relative to the spot where the color was to be matched), and the mean matching result of five observers is indicated. The four panels below each other reflect the four images of Figure 5 from left to right. The small numbers on top of each panel indicate the respective matching location on the stimulus. For illustration purpose, the data are repeated four times (from left to right) to mimic the repetitive, alternating color percepts on the original images (note that colors seen on the reader's monitor may differ slightly from those in our actual experiments).

To provide more insight into the colors that the different observers perceived, Figure 6 shows both the presented colors and the colors as the observers perceived them (note that variations between different color monitors means that what the reader sees in Figure 6 will only approximate to the actual colors we used). The first colored row shows the presented color in the stimulus at the different test locations (1–6). Next, for each condition separately, first the relative positioning of the contours is shown, followed by the averaged perceived colors, as reported in the matching experiment. This figure shows the average response of five observers.

**Figure 7:**
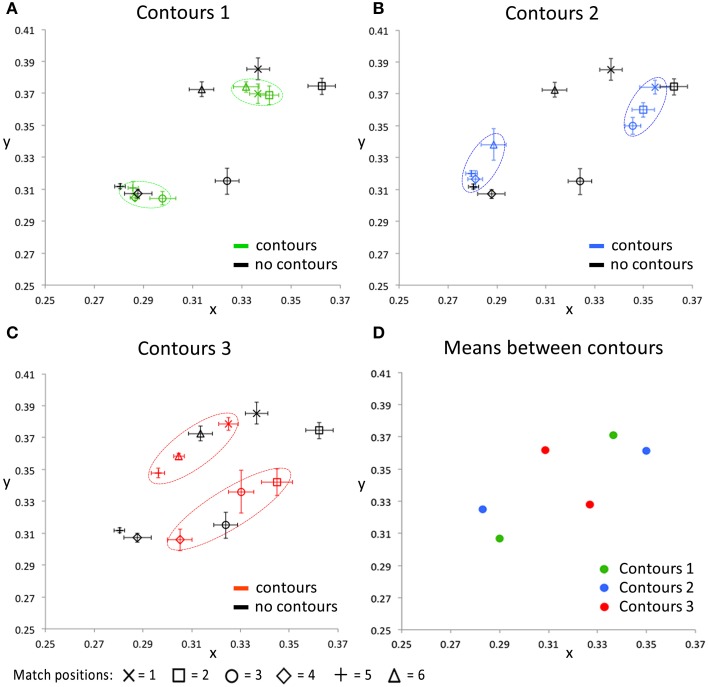
**The CIE xy values for all conditions averaged over all five participants**. The black symbols in **(A–C)** indicate the matching responses on the image without black outlines (Figure 5, right image), for all tested locations, respectively. In addition, each of these 3 panels shows the matching response on one of the contour conditions, for the same tested locations. Error bars represent standard errors (across observers) for the CIE x and y dimensions, respectively. The dashed, colored ovals show the clustering of the color percepts at test locations within the same outlined surface. **(D)** shows the color matching responses, averaged over the three test locations that were presented between the same two contours, for each of the contour conditions separately. Note that the used color-coding in this figure is chosen to make a distinction between the 3 different contour settings and that these colors do not correspond with either the presented or perceived colors directly, in any way.

Figure 7 presents the CIE xy values of the color settings made in this matching experiment. In each of the Figures 7A–C the matches on one of the contour settings is compared with the matches on the image without contours. These plots show that, irrespective of the exact positioning of the contours, presenting the contours brings the colors perceived within each cluster closer together in color space, relative to the colors perceived when no contours were presented. This effect is especially clear for contour settings one and two (Figures 7A,B), where the matches on the six tested locations can clearly be clustered into two groups of three data points (as indicated with the dashed colored ovals). Each of these clusters represents the matches on locations that lie between the same two outlines. For the third contour setting (Figure 7C), a similar effect occurs, though less outstanding. This can be explained by the larger differences between the perceived colors at these tested locations also without contours (as indicated by the black symbols in Figures 7A–C). The perceptual clusters as described above are absent for the condition where no outlines were presented, as indicated by the matches (i.e., the black symbols) being much more spread out over the CIE xy space. Figure 7D summarizes the average matches between each pair of contours, averaged over the three test locations between two outlines. The symmetrical pattern of these data reflects the repetitive gradient in the original colored image. Each contour setting leads to a color percept of two more or less opponent colors (at different sides of a contour), but these perceived near opponent color percepts are different for each contour setting.

## Discussion

In this paper we have demonstrated that a single colored stimulus can produce different percepts depending on the shapes and positions of superimposed thin black contours. The data suggest spatial averaging of the colors between contours. Several studies have demonstrated effects related to spatial averaging. We have already shown spatial spreading of afterimage colors (Van Lier et al., 2009) and also the averaging of “real” colors (Anstis et al., 2012a). The latter effect relates to the phenomenon of monocular rivalry (Breese, 1899) in which perception alternates between two orthogonally oriented spatially overlapping semitransparent near-isoluminant gratings. We previously showed that superimposing black outlines on such a stimulus coinciding with either horizontal or vertical color borders will bias perception toward perceiving a horizontal colored grating or a vertical colored grating, respectively (Anstis et al., 2012a). However, prolonged viewing can cause a perceptual switch to the color grating in the orthogonal orientation, most likely due to adaptation to the first perceived grating and its orientation. The relative instability of this effect could reinforce the argument that the effect is not solely due to low-level color-contour interactions, but is also related to the process of perceptual selection. In our current demonstrations the perceptual effects are more robust: prolonged fixation, controlled horizontal or vertical eye movement and changing the viewing distance does not seem to dramatically change the perceptual outcome. The presence of black contours and their shape and positioning determine color perception. Considering a specific contour arrangement, color perception is constant, not particularly susceptible to effects of adaptation or attention.

The findings we present here add to the demonstrations of contour-dependent and contour-enhanced color perception in the literature. It is likely that our demonstrations share underlying mechanisms with the watercolor illusion (Pinna et al., 2001; Hazenberg and Van Lier, 2013). In addition, we have recently reported demonstrations of color averaging between contours (Anstis et al., 2012a) that are related to the effects we report here. These effects seem to be consistent with what has been called isomorphic filling-in theory (see Von der Heydt et al., 2003), which relies on the idea that color spreads equally in all directions, except across contours. However, the use of relatively complex colored gradients in our demonstrations makes it difficult to relate our quantitative findings to any specific theory on filling in and color contour interactions.

In earlier work on color averaging we additionally showed the role of color contrast induction across contours for afterimages (Van Lier et al., 2009). Anstis et al. (1978) first showed the basic effect of color contrast induction. In our afterimage stimuli, the color of the filled-in surface is not solely determined by the afterimage that followed the color presented within contours but also by the afterimage of the color presented outside the outlined surface, which induces its opponent color on the other side of a luminance border through the process of contrast induction. We speculate that a similar color contrast mechanism may have affected the overall color appearances in our stimuli as well. Further research could test the extent to which the here presented phenomena rely on luminance-defined borders or if other types of contours, such as illusory contours or texture-defined contours could induce similar effects.

Overall, the demonstrations that we have presented here show the versatility of color averaging. The neural mechanisms responsible for this process might not be flexible, but the outcome is flexible, since color perception can change in multiple directions depending on which achromatic information is combined with the presented colors and in which exact spatial configuration. The process of building a reliable, though comprehensible model of the distal world requires many complex neural computations. An important part of this process is object segmentation and surface definition. After surface boundaries are detected, color averaging within the surface could help to minimize perceptual differences within a surface, and thereby enhance relative perceptual differences between different objects and surfaces. In other words, perceptual color averaging could function as a tool to filter out irrelevant stimulus variability from the noisy visual input that generally faces us.

### Conflict of interest statement

The authors declare that the research was conducted in the absence of any commercial or financial relationships that could be construed as a potential conflict of interest.

## Footnotes

^1^This illusion was honored with the second prize at the Illusion of The Year Contest 2014 (http://illusionoftheyear.com/), which contained all demonstrations presented in this manuscript.
